# Mapping Heat Vulnerability Index Based on Different Urbanization Levels in Nebraska, USA

**DOI:** 10.1029/2021GH000478

**Published:** 2021-10-01

**Authors:** Babak Jalalzadeh Fard, Rezaul Mahmood, Michael Hayes, Clinton Rowe, Azar M. Abadi, Martha Shulski, Sharon Medcalf, Rachel Lookadoo, Jesse E. Bell

**Affiliations:** ^1^ Department of Environmental, Agricultural, and Occupational Health College of Public Health University of Nebraska Medical Center Omaha NE USA; ^2^ High Plains Regional Climate Center School of Natural Resources University of Nebraska‐Lincoln Lincoln NE USA; ^3^ Institute of Agriculture and Natural Resources School of Natural Resources University of Nebraska‐Lincoln Lincoln NE USA; ^4^ Department of Earth and Atmospheric Sciences College of Art and Sciences University of Nebraska‐Lincoln Lincoln NE USA; ^5^ Department of Epidemiology Center for Biosecurity, Bio‐preparedness, and Emerging Infectious Diseases College of Public Health University of Nebraska Medical Center Omaha NE USA

**Keywords:** heatwave, heat vulnerability index, factor analysis, environmental health, rural heatwave, Nebraska

## Abstract

Heatwaves cause excess mortality and physiological impacts on humans throughout the world, and climate change will intensify and increase the frequency of heat events. Many adaptation and mitigation studies use spatial distribution of highly vulnerable local populations to inform heat reduction and response plans. However, most available heat vulnerability studies focus on urban areas with high heat intensification by Urban Heat Islands (UHIs). Rural areas encompass different environmental and socioeconomic issues that require alternate analyses of vulnerability. We categorized Nebraska census tracts into four urbanization levels, then conducted factor analyses on each group and captured different patterns of socioeconomic vulnerabilities among resultant Heat Vulnerability Indices (HVIs). While disability is the major component of HVI in two urbanized classes, lower education, and races other than white have higher contributions in HVI for the two rural classes. To account for environmental vulnerability of HVI, we considered different land type combinations for each urban class based on their percentage areas and their differences in heat intensifications. Our results demonstrate different combinations of initial variables in heat vulnerability among urban classes of Nebraska and clustering of high and low heat vulnerable areas within the highest urbanized sections. Less urbanized areas show no spatial clustering of HVI. More studies with separation on urbanization level of residence can give insights into different socioeconomic vulnerability patterns in rural and urban areas, while also identifying changes in environmental variables that better capture heat intensification in rural settings.

## Key Points

- Despite similar incidence rates, Heat Vulnerability Index (HVI) in rural areas is under studied in comparison to urban areas
- The environmental vulnerability variables in rural areas are dissimilar to urban areas, so we applied different variables to calculate them
- We found different organization of socioeconomic variables in calculated HVIs, suggesting separate heat strategies for urbanization levels

## 1 Introduction

Numerous studies suggest that heatwaves cause the highest number of weather‐related mortalities in North America (Braga et al., 2002; Chestnut et al., 1998; Curriero et al., 2002; El Morjanil et al., 2007; Klinenberg, 2015; Mastrangelo et al., 2007; Patz et al., 2000). Hence, the need for understanding these relationships is important due to the continued changes in the frequency, intensity, and duration of heatwaves (Ganguly et al., 2009; Meehl & Tebaldi, 2004; Mishra et al., 2015; Rennie et al., 2019; Stewart & Oke, 2012; Stone, 2007). Specifically, developing local mitigation strategies and adaptation plans are important for reducing casualties and hospitalizations of heatwaves (Ahmed Memon et al., 2008; Bell et al., 2018; Lowe et al., 2011; Rosenfeld et al., 1993; Williams et al., 2019). The goal of mitigation strategies is to reduce the heat exacerbation of Urban Heat Islands (UHI) (Ahmed Memon et al., 2008). UHIs are local urban areas that experience higher temperatures because of the greater storage and more gradual release of heat by pavement, concrete, bricks, and similar materials compared to more natural materials found in surrounding less developed areas (Asaeda et al., 1996; Oke, 2002). A range of mitigation strategies might be employed to reduce UHI effect. These strategies may include using green spaces, light colored pavements or roofs, and increasing vegetation or tree canopies. Adaptation solutions may involve establishing early heat warning systems, designing cooling centers, creating heatwave action plans, and preparing healthcare providers for heatwave events (Boyson et al., 2014; Ebi et al., 2020; Nastar, 2020; Smoyer‐Tomic & Rainham, 2001). Successful research, solution development, and application of solutions require interdisciplinary collaboration among scientists, urban planners, policy makers, health care systems, and communities.

Typically, effective mitigation and adaptation strategies target neighborhoods with the largest temperature intensification and highest number of vulnerable people. Calculating Heat Vulnerability Index (HVI) as a composition of Social Vulnerability Index (SVI), and Environmental Vulnerability Index (EVI) and mapping it over the study region is the preferred method for identifying such vulnerabilities (Méndez‐Lázaro et al., 2018; Reid et al., 2009). Epidemiological research has distinguished several SVI components that are indicators for high risk of heat‐health issues. However, different communities with equal SVI values may experience distinct health outcomes due to changes in heat exposure (Gronlund, 2014; Schwartz, 2005). This variability in vulnerability due to differences in exposure is added to the model by EVIs (Reid et al., 2009). These environmental effects have been considered through a range of variables related to landcover type and population density. Some studies use Normalized Difference Vegetation Index (NDVI) during a heatwave as an EVI proxy, representing the density of green space, to determine different levels of heat intensification (Bradford et al., 2015). Land Surface Temperature (LST) during a heatwave has also been used for this purpose (Johnson et al., 2009; Méndez‐Lázaro et al., 2018). Other studies have used percent imperviousness and/or percent tree canopy as measures of EVI (Conlon et al., 2020). Soil Adjusted Vegetation Index (SAVI), as a proxy for vegetation density, and percentage of land lacking vegetation have also been used in creating EVI (Harlan et al., 2006; Reid et al., 2009). HVI variables can then be defined as linear combinations of SVI and EVI sets and be estimated and mapped within the study area (Reid et al., 2009).

Most studies using HVI mapping focus on urban areas, as higher levels of EVI and population densities lead directly to higher number of poor heat‐health outcomes in urban communities compared to rural. As such, rural areas are overlooked in these types of studies resulting in a barrier to mitigate against health impacts (Kang et al., 2020; Sheridan & Dolney, 2003; Xu et al., 2019). Nebraska is defined by continental climate type with hot summers (Shulski et al., 2015). Days with a high temperature greater than 38°C are more common in the arid west than the humid east. Some years have few high temperature events while extreme summers, such as the warmest on record of 2012, can have greater than 10 days. The multi‐year warm and dry period of the 1930s is anomalous in the climate record (Frankson et al., 2017). Summer minima show a long‐term (since 1895) warming trend while maxima show little change (Shulski et al., 2015).

In recognition of this issue and knowledge of our area of study, Nebraska ‐ being mostly rural and agricultural communities–was classified into urban and non‐urban areas with distinct EVIs, so that HVI variables and the resulting mapping could be developed separately. Subsequently, we assessed the hypothesis that the structure of SVIs in resultant HVIs is different among various urbanization levels, so that considering a range of urban classes for HVI mapping is a necessity for better future planning. We also investigated the potential differences in EVIs that best capture heat intensification levels through land cover type, and finally investigated different potential clustering of high and low vulnerabilities in each urbanization level.

## 2 Materials and Methods

### 2.1 Variable Selection and Data Sources

#### 2.1.1 Socioeconomic Variables

Socioeconomic variables were chosen by conducting a literature review. We specifically looked into works by Johnson et al. (2012), Nayak et al. (2018), Maier et al. (2014), and Reid et al. (2009). These studies have used a range of 6–25 mostly similar demographic variables. We chose seven demographic variables, including: age over 60, age over 60 living alone, below poverty line, race other than white, English language barrier, between 18 and 64 with disability, and education of less than high school diploma. Different epidemiological studies show each of these groups are susceptible to higher degrees of heat vulnerabilities. The elderly group (age >65 years) is usually the foremost group affected, which is due to their lower ability to adapt to extreme weather, as well as their higher rates of preexisting medical conditions compared with other age groups (Curriero et al., 2002; Lin et al., 2009). This situation becomes more challenging for isolated elderly individuals (age >65 years, living alone) who do not have immediate access to help and care during a hazard (such as a heatwave.) English language deficiencies can affect understanding of heatwave warnings, therefore immigrants and groups for whom English is not the first language are more at‐risk (Aubrecht & Özceylan, 2013; Shiu‐Thornton et al., 2007). Disabled populations are also at‐risk because of several reasons: missing warning messages due to vision and hearing impairments or difficulty in relocating to cooling shelters (Abrahamson et al., 2008; Nayak et al., 2018). Poor economic status (measured by the percentage of individuals below the poverty line) is found to reduce the ability of a community to adapt to heatwave events (Curriero et al., 2002; Nayak et al., 2018). In addition, groups with lower levels of education (measured by below high school diplomas for individuals of age 25 or more) have shown to have higher rates of death caused by heatwaves compared to groups with higher levels of education (Medina‐Ramón et al., 2006). Several studies have shown that races other than white are more susceptible to heatwave events and this metric is therefore included in most HVI studies (Gronlund, 2014; O’Neill et al., 2005; Uejio et al., 2011).

We have obtained the data for our selected variables within Nebraska from the American Community Survey five‐year 2012−2016 surveys at census tract level and calculated the ratio of each of these vulnerable groups for each of the 532 census tracts of Nebraska (Burea, 2016).

#### 2.1.2 Urban Categorization and Environmental Variables

Studies have shown the considerable effect of developed areas in exacerbating heatwaves, known as UHI effect (Ahmed Memon et al., 2008). To measure the effect of UHI, researchers use different variables, such as: percent impervious surface, tree canopy percentage, NDVI, population density, or different combinations of them (Bradford et al., 2015; Conlon et al., 2020; Harlan et al., 2013; Reid et al., 2009; Uejio et al., 2011). Most variables are related to the surface types, with a few incorporating population or building density. For this study, we chose the 2016 National Land Cover Database (NLCD) (Dewitz, 2019). NLCD is a map of 30 × 30 m resolution over the United States that distinguishes four different developed land types and 10 different natural land types. Table 1b shows the descriptive statistics of the area percentage of each land type in Nebraska compared to the total area of state and within census tracts.

**Table 1:** Summary Statistics of Variables

(a) Socioeconomic variables					(b) Land cover type variables					
Variable (percent of population)	Mean	sd	Min.	Max.	Land cover type	Area over the state	Percent area over census tracts			
						Total percentage	Mean	sd	Min.	Max.
18–64 years, with disability	9.77	4.47	0.50	38.26	Barren land	0.07	0.14	0.44	0.00	4.67
Low education (below high school diploma for age over 25)	9.24	8.60	0.00	55.33	Cultivated crops	38.18	27.44	30.29	0.00	90.92
Language	1.41	2.86	0.00	26.76	Deciduous crops	1.34	1.84	3.66	0.00	37.04
Over age 60	15.80	6.58	0.00	32.71	Deciduous forest	0.21	12.66	13.04	0.00	75.06
Over age 60, living alone	41.28	13.83	0.00	100.00	Developed high intensity	0.09	5.95	9.66	0.00	84.95
Under poverty level	11.91	8.66	0.00	52.66	Developed open space	2.11	8.32	7.57	0.00	43.13
Race other than White	12.53	14.66	0.00	87.25	Developed low intensity	0.76	24.12	23.06	0.02	82.85
					Emergent herbaceous wetlands	1.93	0.68	1.51	0.00	13.40
					Evergreen forest	0.41	0.06	0.40	0.00	6.58
					Grassland/Herbaceous	52.08	15.48	21.92	0.00	97.65
					Mixed forest	0.14	0.04	0.15	0.00	1.80
					Open water	0.90	1.29	2.91	0.00	33.77
					Pasture/Hay	0.89	0.98	2.02	0.00	18.75
					Shrub/Scrub	0.13	0.04	0.17	0.00	2.22
					Woody wetlands	0.77	0.94	2.25	0.00	31.15

*Note*. (a) Applied socioeconomic variables, as percentage of the vulnerable population within 532 census tracts of Nebraska. (b) Area percentage of 15 different NLCD 2016 land cover types (right) within Nebraska.

Nebraska is considered an agricultural state with a majority of agricultural and grass lands, and few urbanized areas. Therefore, HVIs are not identical for different parts of the state. For this study, we applied and compared two different urban‐rural classification schemes of 2013 National Center for Health Statistics (NCHS)‐county‐level — and the 2010 rural‐urban commuting area (RUCA)‐census‐tract level‐ (Center for Health Statistics, 2013; U.S. DEPARTMENT OF AGRICULTURE, 2020). Subsequently, we used judgment from Nebraskan experts to reclassify the RUCA scheme, considering the best matches with NCHS classes, into a four‐level urban‐rural classification that best suits Nebraska. The NCHS urban‐rural scheme was developed to study the association between urbanization level and health of the residents and is offered on a county level or county‐equivalent entities with six urbanization levels of four metropolitan and two nonmetropolitan classes. RUCA codes classify U.S. census tracts into 10 classes using measures of population density, urbanization, and daily commuting. Nine out of 10 NCHS classes and four out of six RUCA classes are present in Nebraska. To capture the heterogeneity in the sociodemographic, land types, area, and population characters in Nebraska, we opted for the higher resolution tract‐level RUCA urbanization classification. We used Nebraskan expert judgment and regrouped RUCA classes into four groups of “Medium Metro.” “Small Metro.” “Micropolitan.” and “Rural.” to then be used in separate HVI analyses and mappings (Table 2).

**Table 2:** Reclassification of RUCA Over Nebraska Used for This Study

RUCA code	Classification description	Median land area (square miles)	Median population	This study
1	Metropolitan area core: primary flow within an urbanized area (UA)	1.0	3,440	Medium Metro
2	Metropolitan area high commuting: primary flow 30% or more to a UA	15.3	3,965	Small Metro
3	Metropolitan area low commuting: primary flow 10%–30% to a UA			
4	Micropolitan area core: primary flow within an urban cluster of 10,000 to 49,999 (large UC)			
5	Micropolitan high commuting: primary flow 30% or more to a large UC	85.1	3,319	Micropolitan
6	Micropolitan low commuting: primary flow 10%–30% to a large UC			
7	Small town core: primary flow within an urban cluster of 2,500 to 9,999 (small UC)			
8	Small town high commuting: primary flow 30% or more to a small UC			
9	Small town low commuting: primary flow 10%–30% to a small UC	–	–	Not in Nebraska
10	Rural areas: primary flow to a tract outside a UA or UC	439.9	2538	Rural

We used percentage area of the four developed land cover types in NLCD — developed open space, developed low density, developed medium density, and developed high density‐within each census tract to differentiate levels of heat exacerbation. We then calculated their summary statistics within each of four urban classes of Nebraska. If the maximum percentage area is below 10% for all developed types, we switched to grouped land types with similar summer NDVI values (Eastman et al., 2013). In this case the choice of 10% is an arbitrary threshold below which we consider the effect of heat exacerbation by urban landcover types to be negligible and happens only in the vicinity of paved roads that are unlikely to affect residents. Previous studies show that NDVI can also be used as an indicator for LST and UHI effects (Yue et al., 2007). Different land types can also be grouped into four classes based on their summer NDVI values (Kong et al., 2016). We considered these four NDVI based classifications as follows: Class1 (consists of the four developed land types and Barren Land), Class 2 (includes Deciduous Forest, Evergreen Forest, Mixed Forest, and Cultivated Crops), Class 3 (composed of Shrub/Scrub, Grassland/Herbaceous, and Pasture/Hay), and Class 4 (includes Woody Wetlands and Open Water). These groupings represent landcover types with most similar summer NDVI values within a group and considerable differences among different groups (Kong et al., 2016). For tracts with scarcities of developed land percentage, we used the land percentage of any of the constructed land classes that had above 10% maximum in tract areas.

### 2.2 Factor Analysis With Varimax Rotation to Create HVIs

Exploratory Factor Analysis (EFA) was used to extract HVIs as the underlying unobserved variables to capture the covariance structure of our observed socioeconomic and environmental vulnerability variables (Costello & Osborne, 2005). We first standardized all data sets into ratios containing values between zero and one. Four data matrices were created–one for each urban type–with different land type classes as explained before. Parallel analysis on each matrix suggested the number of factors that can adequately capture the covariance structure among observed variables (Horn, 1965; Revelle, 2017). The initial results of EFA were then rotated using varimax rotation to improve the interpretation of results by simplifying the HVIs through redistribution and separation of initial variables among different resulted factors.

### 2.3 Mapping HVI Values

For mapping HVI, we categorized values into five ordinal groups. To find appropriate break values for classifications, we calculated absolute deviation around class medians (ADCM) for five mostly used classification methods for creating choropleth maps (Coulson, 1987). ADCM provides a comparison variable of alternative classifiers for the same value of number of levels (k). We subsequently chose the classification method of minimum ADCM value and changed each factor score into its vulnerability level (a value between 1 and 5), with 1 representing the lowest and 5 the highest vulnerability. Then ArcGIS Pro software was used to create choropleth maps of vulnerability factors with five levels (Corbin, 2015). In the next step, we calculated total vulnerabilities by summing up the ordinal values of different factors in each census tract.

### 2.4 Cluster and Outlier Analysis of Total Vulnerability Values

Local Indicators of Spatial Association (LISA) was used to analyze the hotspots, cold spots, and outlier census tracts of total vulnerabilities in each urban class of Nebraska (Anselin, 1995). This method compares the difference of the desired variable in each tract with its neighboring tracts with a distribution of permutations of randomly assigned values to the tracts. We used Anselin Local Moran's I analysis in ArcGIS Pro software with 500 permutations and significance level of 0.95.

## 3 Results

More than 90% of total land area in Nebraska consists of Grassland or Cultivated Crops (Table 1). While total percentages of the four developed land types (NLCD, 2016) contains 3.17% of total land, their tract level distribution shows a maximum and mean percentages of 43.14% and 8.32% for Open Space; 82.85% and 24.12% for Low Density; 75.06% and 12.66% for Medium Density; and 84.95% and 5.95% for High Density. This is the result of high concentration of urban areas in a few small regions of the state and shows the necessity of grouping these tracts into groups with similar urbanization levels.

**Figure 1:**
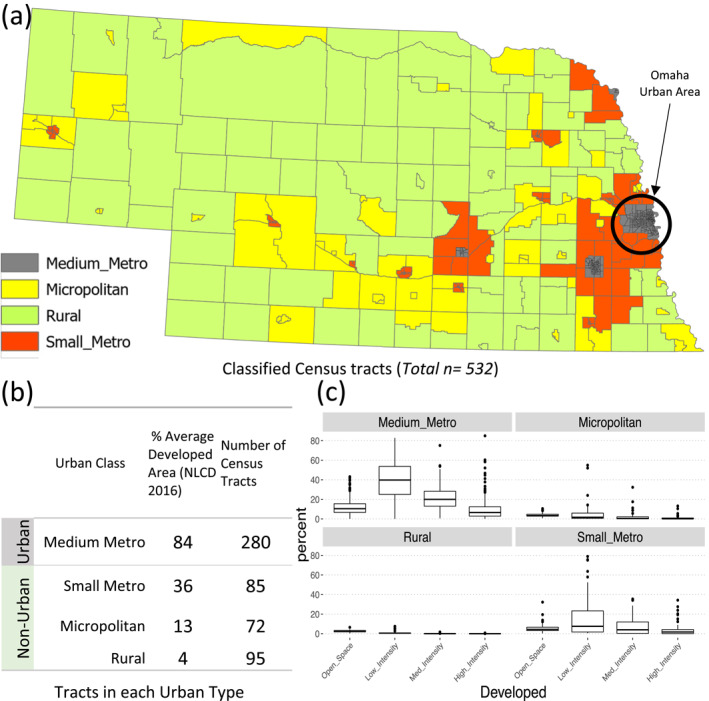
Classification of Nebraska’s tracts (a) The four classes of urban type considered for the Environmental Vulnerability Index mapping of Nebraska. (b) The number of tracts in each considered class, and average developed area, (c) Boxplots of percentages of the four types of developed land types in each of the considered classes.

**Figure 2:**
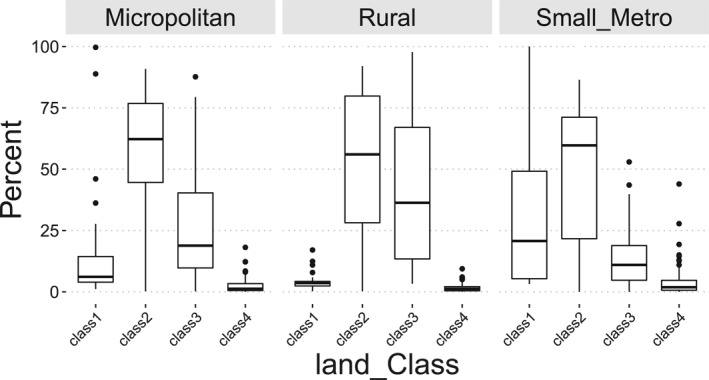
The distribution of area percentage of four land type classes in three Non‐Urban groups of tracts (Figure 1). Classes are composed of land cover types in National Land Cover Database 2016 that have similar Normalized Difference Vegetation Index summer values. Class1 consists of the four developed land types and Barren Land. Class2 includes Deciduous Forest, Evergreen Forest, Mixed Forest, and Cultivated Crops. Class3 consists of Shrub/Scrub, Grassland/Herbaceous, and Pasture/Hay. Finally, Class4 includes Woody Wetlands, and Open Water.

We reconfigured the RUCA tracts in Nebraska to combine classes (Table 2). The revised RUCA classification renders 280, 85, 72, and 95 census tracts into Medium Metro, Small Metro, Micropolitan and Rural classes, respectively (Figure 1b). Medium Metro, Small Metro, Micropolitan and Rural classes had median areas of 2.59, 39.6, 220.46, and 1139.59 square kilometers and median populations of 3,734, 3,996, 3,441, and 2469, respectively. While only about 18% of tracts are considered as rural in our classification (95 out of 532 as in Figure 1b), they contain 73% of total area and 13% of total population. The largest tracts population resides in Medium Metro section with 56% of total population (ACS 5‐year 2016) while it contains 0.85% of total land area. The same transition can be tracked in the percentage of urban land types (Figure 1c). Medium Metro consists of the highest percentages of all developed land types that gradually decrease in Small Metro, Micropolitan, and Rural tracts (Figure 1c), from an average of 84% of total developed areas in Medium Metro to 4% in Rural areas. We, therefore, used combinations of the four NDVI‐based classes as our environmental variables for Small Metro, Micropolitan, and Rural groups of tracts. Figure 2 shows the percentages of the four NDVI‐based classes in three Non‐Urban groups (Figure 1b). We distinguish a gradual shift from highest developed land types (class1) in Small Metro tracts to the lowest in Rural types (Figure 2). In construction of input matrices for EFA, we added class1 and class2 for the Small Metro tracts, therefore ending with a total of nine observable variables (seven socioeconomic and two environmental). From Figure 2, class1 and class4 land types have a very small share of land area in Micropolitan and Rural areas, we therefore included percentages of class2 and class3 land types in each census tracts as the environmental variables in the respective data matrices.

**Figure 3:**
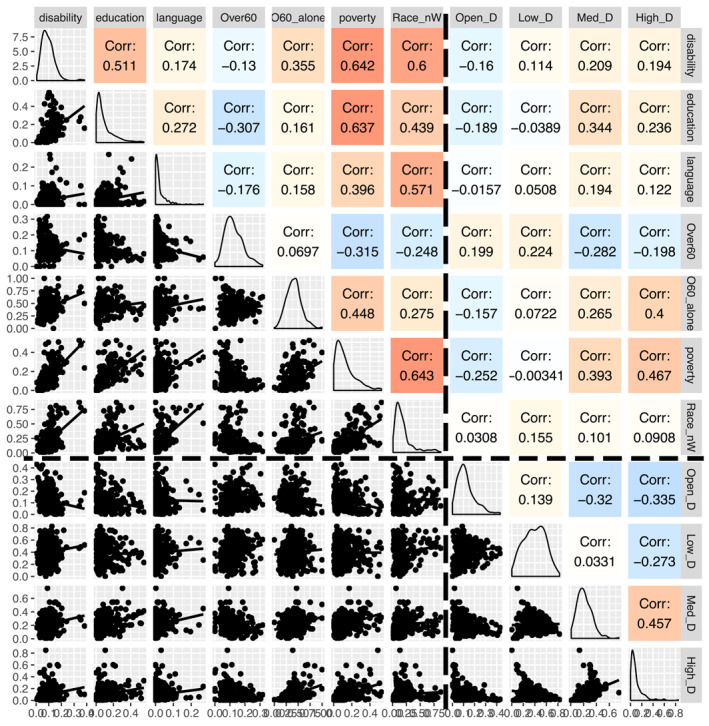
Exploratory data plots for variables used in Heat Vulnerability Index analysis of Medium Metro tracts (*n* = 280). Below the diagonal show scatterplots of each variables. Above the diagonal presents the correlation values, and the density plots are on the diagonal. Top left box contains sociodemographic variables. Bottom‐right box contains considered environmental factors, and the other two box areas contain plots of socio‐demographic versus environmental variables.

**Figure 4:**
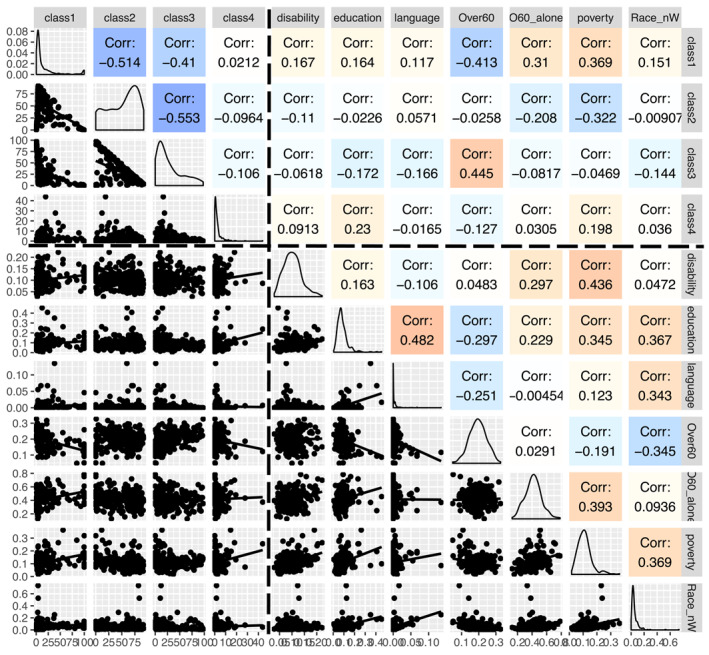
Same as Figure 3, but for Non‐urban tracts (*n* = 252 tracts). Upper left boxes contain environmental variables, lower right boxes are socioeconomic variables, and the two other boxes contain information of socioeconomic‐environmental interactions.

Pairplots of socioeconomic and environmental variables for urban and non‐urban areas show considerable difference in the correlations (Figures 3 and 4). There is a high correlation among poverty, race other than white, education and language in Medium metro tracts, while this was not seen for the three non‐urban areas (Figure 4). The ordering of these values, however, is nearly similar with poverty‐disability with highest correlation values.

**Figure 5:**
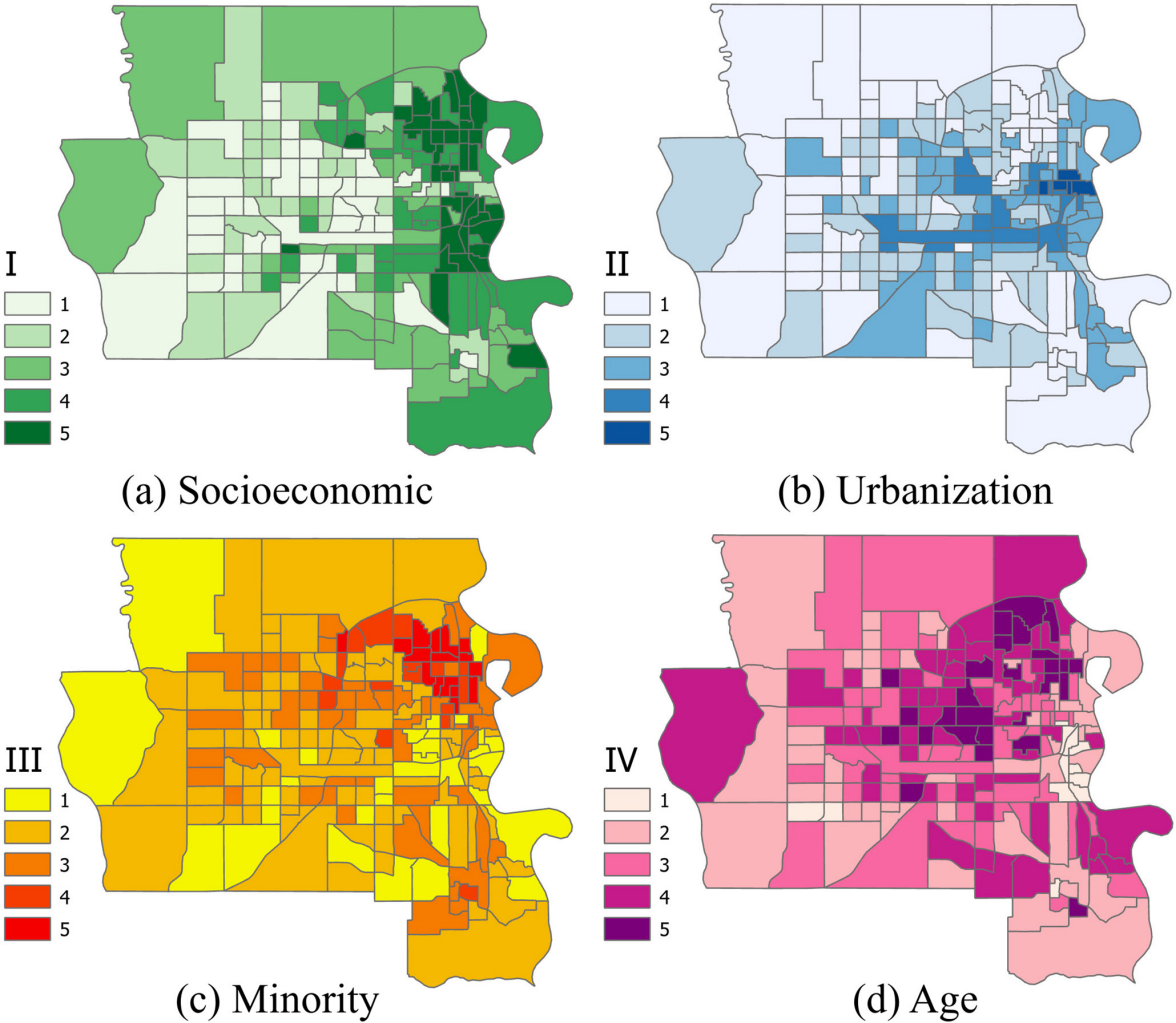
Map of four vulnerability mappings of Omaha urban area as part of the Medium Metro classification (zoomed out of Figure 1a) (*n* = 196). Four factors of vulnerabilities are named based on the variables that highly participate in each factor loading (Table 3). Higher values represent higher vulnerabilities.

The results of factor analyses show differences in the combinations and coefficients of variables for each urban classification (Tables 3 and 4). Medium Metro area and Rural areas are captured by four factors, while parallel analysis shows the adequacy of three factors for the other two urban types. Medium Metro contains the most populated and developed areas of Nebraska. The first factor explains 18% of the variation that is mostly defined by socioeconomic variables (Figure 5a). The four variables in this factor generally show highest correlations in Figure 2. The second factor in Medium Metro areas contains the two higher density developed land types (High Density and Medium Density percentage), and the percent of age over 60, living alone. The total variance explained by these four factors are 0.53 (Table 3). In Small Metro area, three factors capture 59% of the variability of observed variables, with the most important factor being the two land type classes (Table 3). The second factor captures three of the socioeconomic factors with highest loading of disability. The three captured variables seem related, therefore finding them in one factor seems to be reasonable, although the same variables do not contain in the same factor for the case of Medium Metro. The last factor for Small Metro areas captures education, language, and race other than white. (Table 3). The first factor in Micropolitan group (Table 4) captures 20% of the variation and is loaded highly on four related socioeconomic variables: education, language, and race show nearly similar coefficients (0.67, 0.64, and 0.67 respectively) while Over60 has a less value. But compared to the two more urbanized categories Over60 is opposite to other socioeconomic variables captured by this factor. This is in contrast with Medium Metro and Small Metro (Table 3), but in line with Rural areas (Table 4). Here we have captured a change of pattern in the relationship of Over60 with other socioeconomic variables while urbanization category changes. Land cover types of class2 and class3 load highly in second factor of Micropolitan group (Table 4) and the last factor is made of the remaining socioeconomic variables (disability, Over60 and alone, and poverty). Four factors capture 63% of total data variation in Rural areas (Table 4). Although, the third factor contains only one observed variable with a very high correlation of 0.99. Table S1 in Supporting Information S1 shows the result of an EFA analysis with all tracts involved and the percentages of four developed land types as EVIs. It clearly shows how urban areas have overshadowed less urbanized areas, as the first factor is highly dependent on two of developed land types.

**Table 3:** Results of Factor Analysis for Medium Metro and Small Metro Urban Types

Factors	I	II	III	IV		I	II	III
Disability	***0.81***	0.18	0.13	0.14	Class1/Land	***0.92***	0.21	
Low education	***0.60***	0.20	0.22	−0.26	Class2/Land	***−0.93***	−0.20	
Language	0.12		***0.68***		disability		***0.89***	
Over 60	−0.20	−0.10	−0.20	***0.60***	Low education		0.29	***0.73***
Over60/alone	0.22	***0.56***	0.17	0.36	language		−0.18	***0.78***
below poverty	***0.66***	0.46	0.40		Over 60	−0.27		−0.30
Race/noWhite	***0.56***		***0.75***		Over60/alone	0.35	***0.59***	
Developed/Open	−0.15	−0.40		0.24	below poverty	0.30	***0.60***	0.31
Developed/Low	0.12	−0.16		0.35	Race/noWhite		0.13	***0.50***
Developed/Med	0.19	***0.50***	0.10	−0.22				
Developed/High		***0.78***		−0.19				

Medium metro (*n* = 280)					Small metro (*n* = 85)		
					I	II	III
SS loadings	1.94	1.66	1.35	0.86	1.99	1.71	1.59
Proportion Var	0.18	0.15	0.12	0.08	0.22	0.19	0.18
Cumulative Var	0.18	0.33	0.45	0.53	0.22	0.41	0.59

*Note*. Top tables show the factor loadings of each variable. Bottom tables contain sum of the squared loadings on first row (SS loading), The variance captured by each factor in the second row, and cumulative captured variances in the third row. Absolute values > 0.49 are the most significant loadings on that factor. All models show *p*‐value < 0.0001 for model‐based χ^2^ test.

**Table 4:** Same as Table 3, But for Micropolitan and Rural Urban Types

	I	II	III		I	II	III	IV
Class2/Land		***−0.86***	−0.25	Class2/Land	***−0.97***	0.21		
Class3/Land		***0.92***	−0.23	Class3/Land	***0.98***	−0.19		
Disability	−0.16		***0.54***	disability				***0.54***
Low education	***0.67***		0.16	Low education	−0.12	***0.52***	0.27	
language	***0.64***			language		0.31		
Over 60	***−0.57***		−0.12	Over 60	0.27	***−0.57***	0.18	0.31
Over60/alone	0.23		***0.54***	Over60/alone		0.11	***0.99***	
below poverty	0.30		***0.72***	below poverty	0.36	0.41	0.12	***0.78***
Race/noWhite	***0.67***	0.22		Race/noWhite		***0.64***		0.29

Micropolitan (*n* = 72)				Rural (*n* = 95)			
				I	II	III	IV
SS loadings	1.79	1.65	1.26	2.13	1.36	1.10	1.09
Proportion Var	0.20	0.18	0.14	0.24	0.15	0.12	0.12
Cumulative Var	0.20	0.38	0.52	0.24	0.39	0.51	0.63

The results of the highest urbanized areas were mapped separately for clarity. Mapping values of each HVI for each tract shows patterns of high vulnerabilities in Omaha area (Figure 5). Vulnerabilities are categorized from lowest to highest on a scale from 1 to 5. The highest vulnerable tracts within Greater Omaha area are concentrated in east central, near the border with Iowa (Figure 5). Distribution of Socioeconomic HVI variable (Figure 5a) shows a clear concentration of high vulnerability in the eastern side of the state compared to the west. While for urbanization (Figure 5b), this concentration is mostly around a central east‐west line in the east half of area. A nearly similar pattern can be recognized for Age variable (Figure 5d). The variable representing minority populations is more toward north‐east tracts.

**Figure 6:**
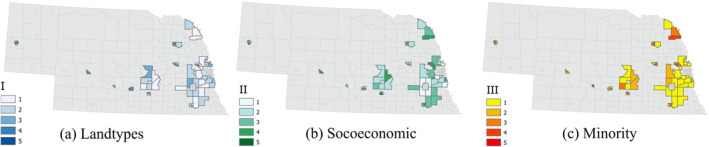
Map of three Heat Vulnerability Index variables for Small Metro group of census tracts.

**Figure 7:**
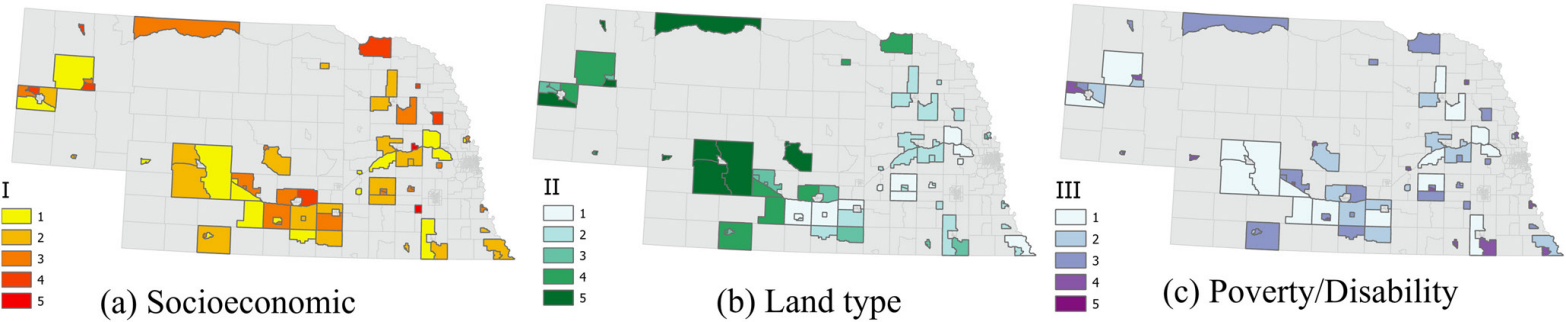
Heat Vulnerability Index levels in Micropolitan tracts (*n* = 72).

**Figure 8:**
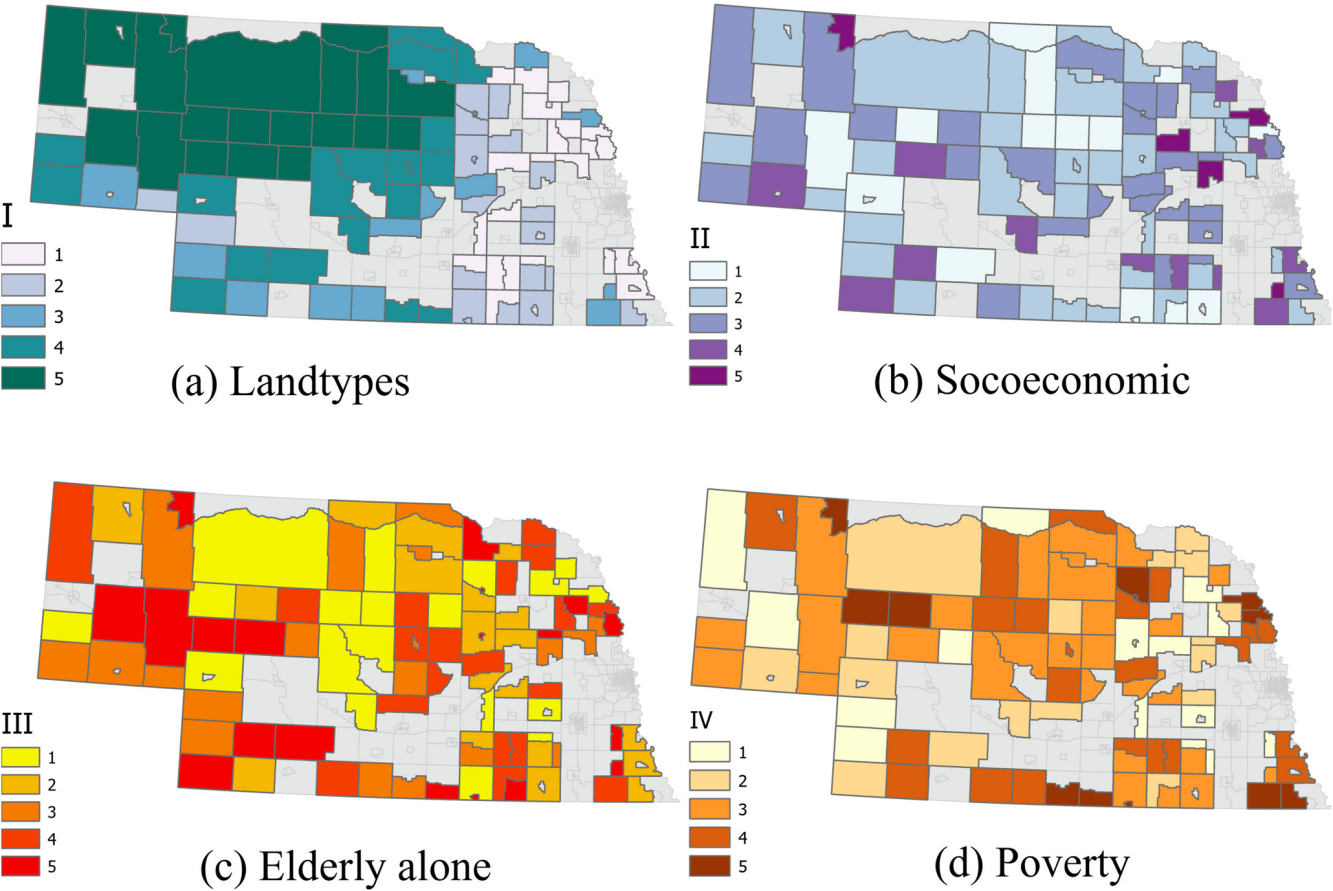
Heat Vulnerability Index variable distribution for Rural tracts (*n* = 95).

The spatial distribution of three HVI values in Small Metro tracts do not appear to follow any specific pattern and are not similar in any of the three (Figure 6). Even areas closer to Medium Metro group of tracts are not high in any of the three factors. However, the smaller and more urbanized tracts generally show higher vulnerability levels in all three factors. This can be an indication of the transition from more into less urbanized tracts within this group. The distribution of three vulnerability factors in Micropolitan areas do not show a specific concentration of low or high values (Figure 7). The socioeconomic factor (Figure 7a) has its highest vulnerable area in northeast Nebraska and southcentral Nebraska north of the Platte River. HVI values seem to be randomly distributed for all three SVI related variables in Rural tracts (Figure 8). The first ‐and most prominent‐ HVI (Figure 8a) is related to the land cover type and has the highest level of vulnerability concentrated on the northwest part of the state. This is where there are grassland areas as included in our class3 group of land covers. Compared to class2, with croplands as its major landcover type, this group represents lower NDVIs, therefore higher values of LST and more intensification of heat.

**Figure 9:**
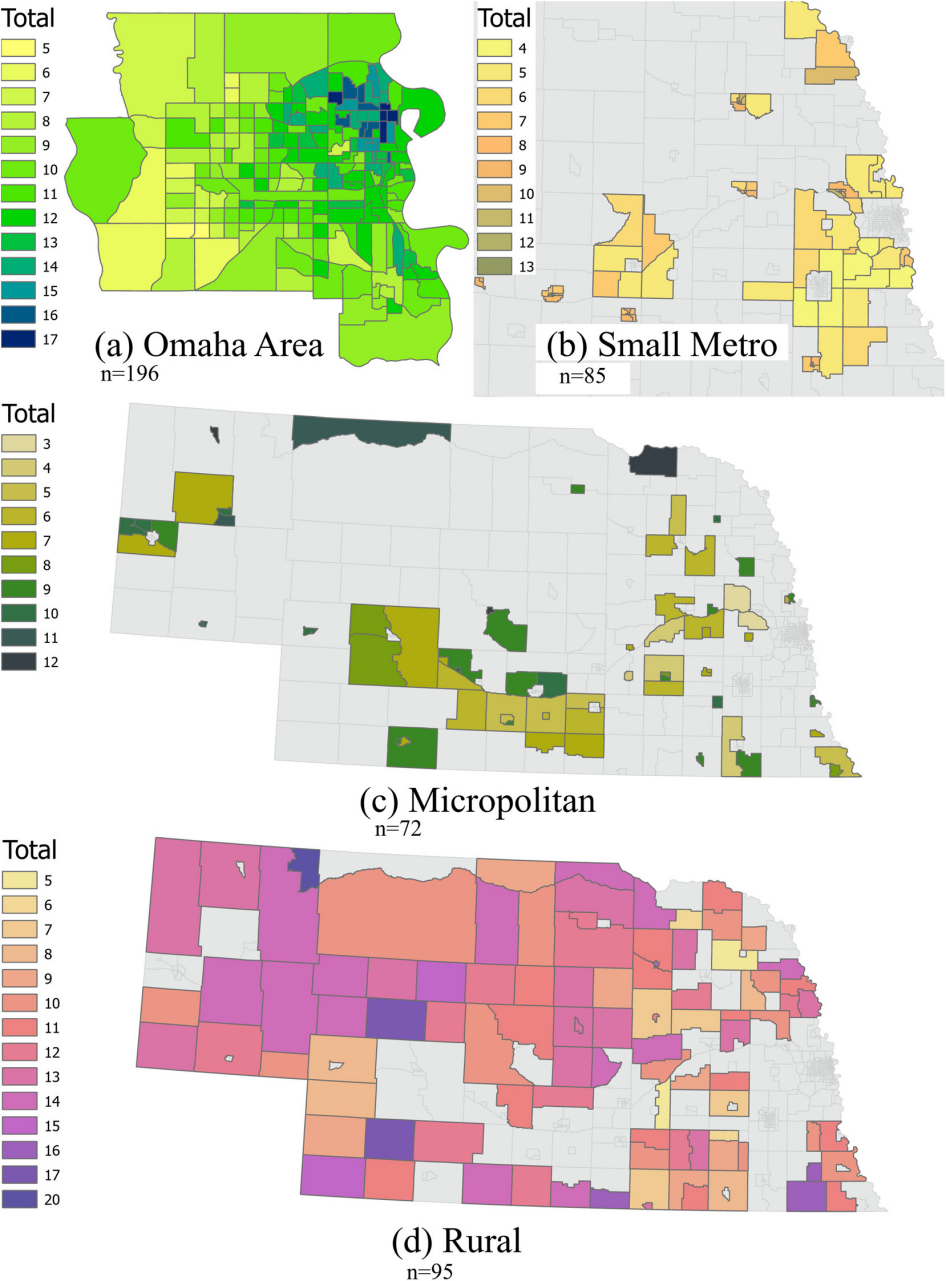
Total Heat Vulnerability Index level values in each of the four urban classes. (a) Omaha area as the most populated Medium Metropolitan area. (b) Part of Small Metro area (Eastern Nebraska as in Figure 1a). (c)Micropolitan areas. (d) Rural areas.

Figure 9 depicts spatial patterns of Total HVI in four urban classes. In the Omaha area (Figure 9a), we see a clear radial pattern starting with the highest values in the eastern part of the city. Considering the four HVIs with possible values of 1–5 for each, the total vulnerability can be a value between 4 and 25. No single tract is least vulnerable in all four factors, nor do any have the highest value in all factors. The minimum total vulnerability is 5 for several of the outer tracts, and the maximum is 17 for 5 tracts around the downtown area of Omaha. Total HVI values in Small Metro group do not show specific patterns (Figure 9b), following the similar trend in three individual HVIs (Figure 6). The possible range of total vulnerability values can be 4 to 20 in Medium Metro and Rural Areas, and for Small Metro and Micropolitan groups these values can be 3 and 15. The two highest vulnerable tracts are on the northeast section of the state. Considering three vulnerability factors with levels of 1–5, the total vulnerability can be a value between 3 and 15. There are 2 tracts in the east side of this area (immediately west of Omaha area) that have a total vulnerability of 3. It means that they fall into the lowest vulnerable groups in all three categories for Micropolitan areas. Rural areas seem to be randomly distributed in different parts of the state. Western Nebraska seems more toward higher values compared to the east. This can potentially be attributed to the effect of first vulnerability factor (Figure 8a). Considering four vulnerability factors with possible ranges of 1–5 in this urban class, a minimum of 4 and maximum of 25 is expected for these tracts which has not occurred in any of the tracts in this group. No tract is lowest or highest in all vulnerability categories in this urban class. Figure S1 in Supporting Information S1 shows the resulting map of total vulnerabilities from a “one solution fit all model." It illustrates that all high vulnerabilities are just focused on the highest urbanized areas and rural areas are overlooked.

**Figure 10:**
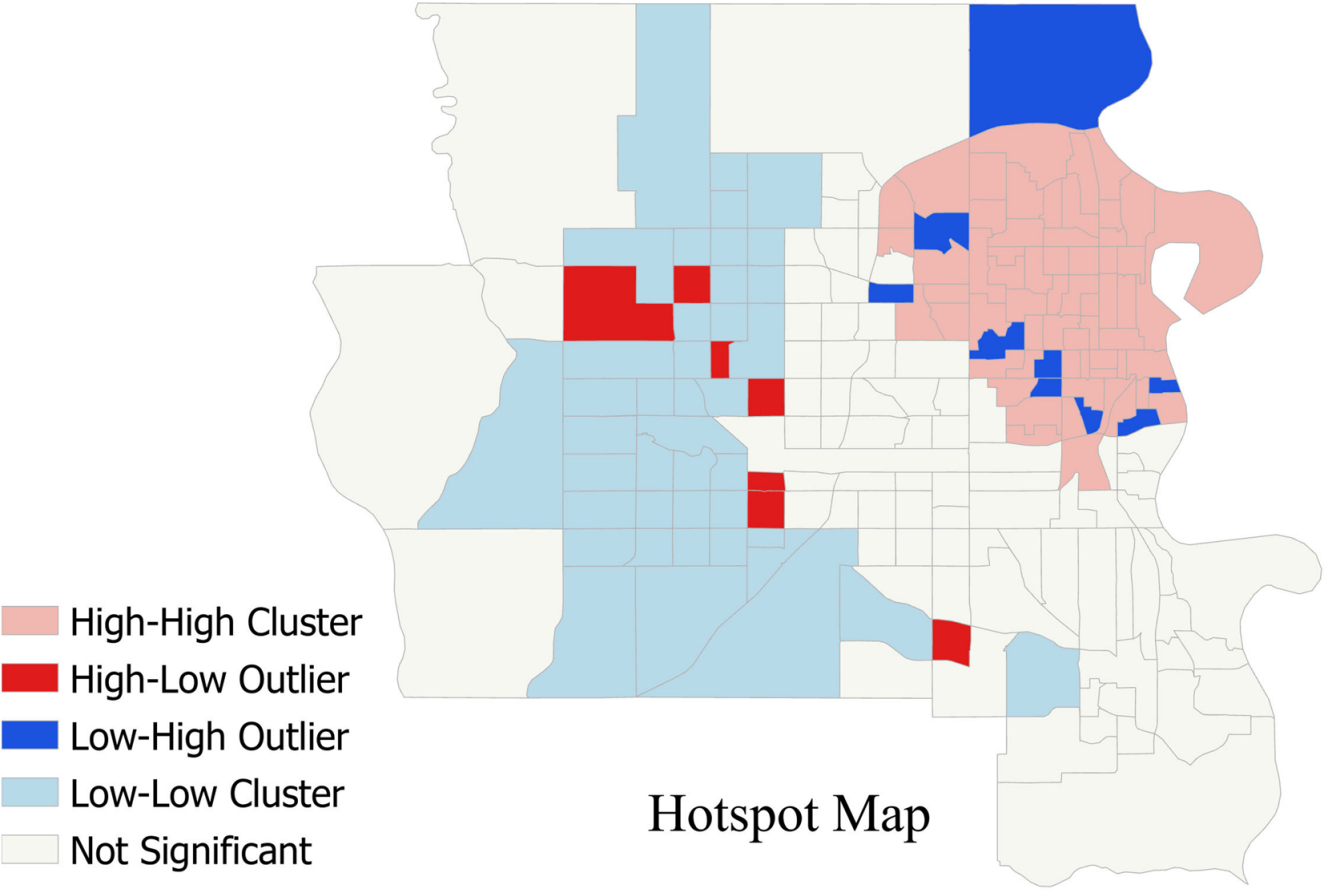
Cluster and Outlier analysis of Total Vulnerabilities for Omaha Area (zoomed from Figure 1a). In part (b) instead of Moran’s scatter plot include Rural areas.

The results of LISA analysis for Omaha area shows clusters of High‐High and Low‐Low total HVI (Figure 10). This could be expected from similar patterns of individual HVIs, then leading into a radial pattern of concentrated high HVIs in Figure 9.

LISA analysis for Rural class did not show any significant classification of clustering or outlier that could be expected from Figure 9d. For other two groups (Small Metro and Micropolitan), we did not consider LISA analysis useful, due to the scattered and disconnected groups of tracts without any pattern in their distribution. Therefore, distinguishing any clustering or outlier tracts can be misleading.

## 4 Discussion

To our knowledge, this is the first study to show that separating census tracts into different categories based on urbanization levels results in different combinations of socioeconomic variables in calculated HVIs. For example, disability contributes highest in HVI factors in both more urbanized groups‐ Medium Metro and Small Metro‐, while it only appears in the last HVI factors for the two higher rural areas–Micropolitan and Rural with the lowest coefficients compared to other variables. On the other hand, race other than white is the most contributing socioeconomic variable in two higher rural areas, which is not the case for two more urbanized classes. We also found that both the structure of land cover types used as environmental variables–and their contributions in HVI factors vary for different urban classes, suggesting different potential mitigation strategies for each group. And finally, our hotspot analysis showed clusters of high HVI and low HVI concentration in the highest urban level class, but no such pattern was distinguished in the other three classes.

Reid et al. (2009) suggest that different regions experience different levels of vulnerability with highest vulnerabilities in the most populated urban areas. Our study confirms this finding and extends these differences into the combination of the initial variables in final HVI factors. To measure environmental vulnerability through heat intensification, different studies include measures of lack of green spaces, or the abundance of impervious surfaces, or building intensity in their HVI model (Bradford et al., 2015; Nayak et al., 2018; Reid et al., 2009). A large part of our study area mostly contains crop and grassland land cover types that are categorized as green areas in mentioned studies. These land cover types dominate the two higher rural areas. For our study, we have used summer NDVI differences of these land types as surrogates to differentiate their LST, therefore grouping them based on their different levels of heat intensification. Previous studies show that there is a negative correlation between summer NDVI and LST, however this correlation varies by season and region (e.g., Kaufmann et al., 2003; Marzban et al., 2018). We therefore suggest that a future study focusing on this relationship within Nebraska may increase the accuracy of our environmental vulnerability variables for rural areas.

We suspect that rural areas may contain socioeconomic variables that were used in a limited fashion in similar studies focusing on urban areas. For example, we investigated the percentage of outdoor workers in our different urban types but could not find any significant differences, therefore we did not include it in our study. Another example can be inclusion of tribal communities as sources of increased adaptive capacities due to the support they provide to their vulnerable population in rural areas that include such social ties.

Sheridan and Dolney (2003) found comparable mortality rates in suburban and rural areas of Ohio to its urban areas, and Maier et al. (2014) found that half the counties with highest HVI in Georgia are rural. As a future step to the current study, after acquiring related health data, we suggest verifying these maps with related mortality and morbidity levels in Nebraska. Heatwaves Early Warning Systems (HEWS) are among top priorities in heatwave preparation plans in different countries (Lowe et al., 2011; Matzarakis et al., 2020). The results of this study can increase the effectiveness of regional HEWS system for Nebraska through informing communication and dissemination strategies, as well as recommended prevention strategies. Communication and dissemination of information should be tailored to the target audiences at the local level. Prevention strategies such as HEWS can include targeted infrastructure, to ensure transportation to cooling facilities, by targeting the identified most vulnerable audiences. For longer term planning, projected population change and potential urban development plans needs to be implemented in the presented framework with considerations for uncertainty quantification for more accurate, future informed long‐term planning.

## 5 Conclusion

Heatwaves are expected to become increasingly dangerous for human health. Many studies focus on the effect of heatwaves in urban areas or use one model to map related vulnerabilities in urban areas and their surrounding less urbanized regions. We showed that separating heterogeneous study areas into different groups can reveal different structures of socioeconomic variables in the development of HVIs, so that including them in one model may be misleading and neglecting less urbanized areas. In our analysis, we have considered these groups based on their urbanization levels and the expert suggestions on how they can fit into groups. These results can better help decision makers at various levels to focus on customized solutions for each urbanization level of residence. This study focuses on Nebraska as a state with large rural areas and a small percentage of urban systems. We suggest that similar frameworks can be applied to other regions that contain similar heterogeneity.

## Conflict of Interest

The authors declare no conflicts of interest relevant to this study.

## Supporting information

Supporting Information S1

## Data Availability

The data that support the findings of this study are openly available in Mendeley Data at doi: 10.17632/79mg69dz98.2 (Jalalzadeh Fard, 2021).
