# Supplementary material for: Mapping Heat Vulnerability Index Based on Different Urbanization Levels in Nebraska, USA
Source: Geohealth. 2021 Oct 1;5(10):e2021GH000478. doi: 10.1029/2021GH000478 (PMC8533801; doi:10.1029/2021GH000478)
Supplement: Supplementary file 1 — Supporting Information S1 [file GH2-5-e2021GH000478-s001.pdf]

Geohealth

Supporting Information for

Mapping Heat Vulnerability Index Based on Different Urbanization Levels in Nebraska, USA

Babak J.Fard[1], Rezaul Mahmood[2], Michael Hayes[3] , Clinton Rowe[4], Azar M.Abadi[1], Martha Shulski[2], Sharon Medcalf[5], Rachel Lookadoo[5], and Jesse E. Bell[1,2]

[1]Department of Environmental, Agricultural, and Occupational Health, College of Public Health, University of Nebraska Medical Center.

[2]High Plains Regional Climate Center, School of Natural Resources, University of Nebraska-Lincoln.

[3]Institute of Agriculture and Natural Resources, School of Natural Resources, University of Nebraska-Lincoln.

[4]Department of Earth and Atmospheric Sciences, College of Art and Sciences, University of Nebraska-Lincoln.

[5]Department of Epidemiology, Center for Biosecurity, Bio-preparedness, and Emerging Infectious Diseases, College of Public Health, University of Nebraska Medical Center.

Contents of this file

Figures S1 to S2
Table S1

Factors -->	I	II	III	IV
disability		0.23	***0.65***	
Low education	0.25	0.43	0.38	-0.19
language	0.19	***0.58***	0.10	0.20
Over 60	***-0.53***	-0.46	0.11	-0.14
Over60/alone	0.12		***0.60***	
below poverty	0.27	0.46	***0.70***	-0.14
Race/noWhite		***0.81***	0.32	0.35
Developed/Open		0.15	-0.17	***0.61***
Developed/Low	0.37	0.13		***0.72***
Developed/Med	**0.87**	0.16	0.13	0.37
Developed/High	**0.66**	0.13	0.27	

SS loadings	1.80	1.74	1.66	1.27
Proportion Var	0.16	0.16	0.15	0.12
Cumulative Var	0.16	0.32	0.47	0.59

All Tracts (n=532)

Table S1. Results of factor analysis for combination of all tracts. Top table shows the factor loadings of each variable. Bottom table contains the sum of the squared loadings on first row (SS loading), The variance captured by each factor in the second row, and cumulative captured variances in the third row

.

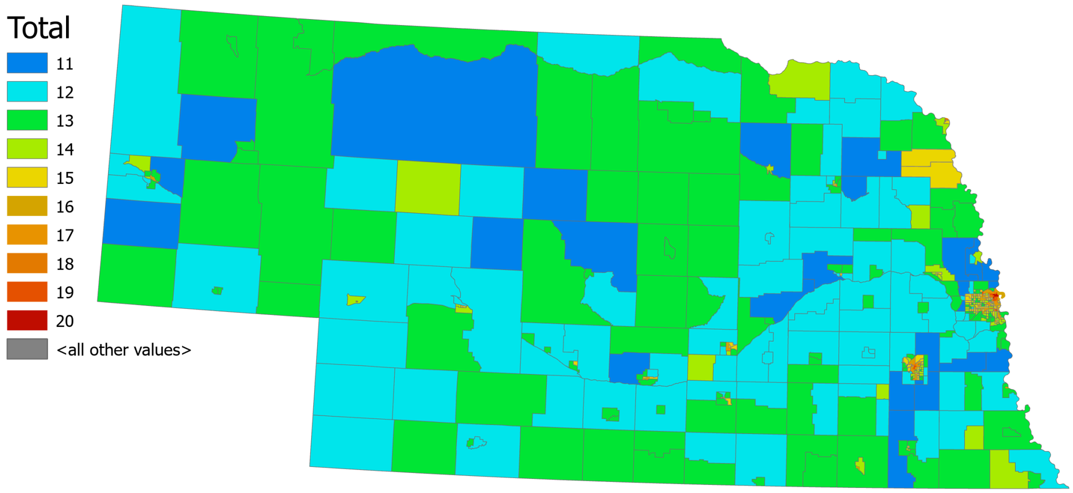

Figure S1. Total vulnerability map resulting from one fit all solution. All highly vulnerable areas- from score 17 to 20- are located within the highest urbanized tracts (Medium Metro group in Figure 1).

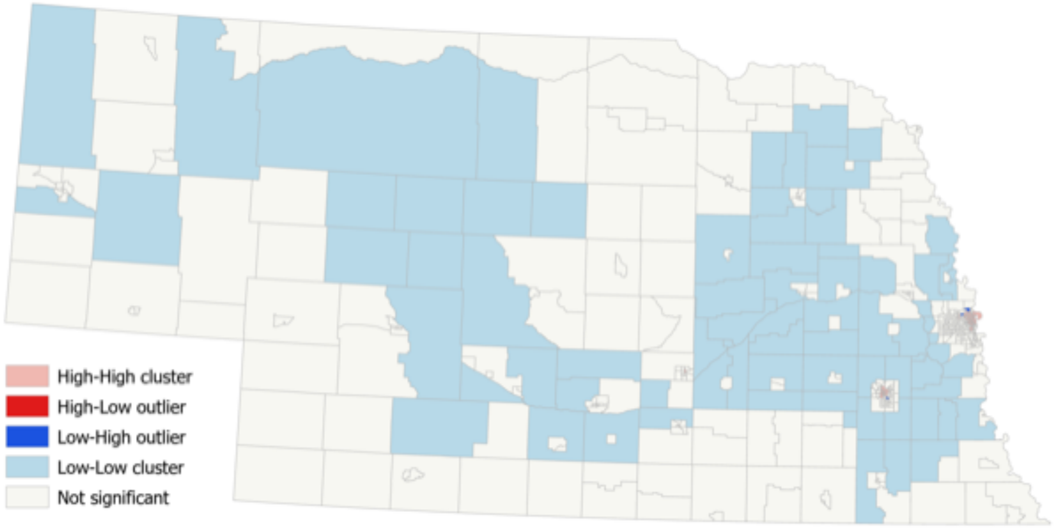

Figure S2. Total vulnerability map resulting from one fit all solution. All highly vulnerable areas- from score 17 to 20- are located within the highest urbanized tracts (Medium Metro group in Figure 1).
